# Myopathy

**Published:** 1899-06-17

**Authors:** 


### MYOPATHY.

In a lecture on cases of myopathy recently delivered at the National Hospital for the Paralysed and Epileptic, Dr. C. E. Beevor,[1] said that these cases (in which the muscles themselves are at fault, the spinal cord and the peripheral nerves being healthy) might be divided into three groups: (1) Pseudo-hypertrophic paralysis, which generally begins in early childhood, though it may begin at puberty, and in which the great point is usually the hypertrophy or enlargement of the muscles. The muscles most commonly affected are those of the calf, the extensors of the knee, and the glutei. Certain muscles in the upper extremity are also affected. Sometimes atrophy occurs without preceding hypertrophy. (2) The juvenile form described by Erb, in which the onset is about puberty, or from puberty to 20 years of age. As a rule there is no hypertrophy in this disease, and the muscles which are atrophied are different from those affected in pseudo-hypertrophic paralysis. The whole of the trapezius is affected, and the biceps, the supinator longus, the triceps, the thigh muscles, the anterior tibials, and the peronei are atrophied, the muscles which are preserved being usually the deltoid and the supra- and infra-spinati. Neither the face nor the forearms are affected. (3) Finally, we come to the facio-scapulo-humeral type of Landouzy and Déjerine, which differs from the other two in that the face is affected. The muscles which are most involved are the orbicularis oris, levator labii superioris, and orbicularis palpebrarum. The patient is generally unable to whistle or to elevate the upper lip, or to close the eyes completely. Otherwise the muscles affected are those mentioned in regard to the previous form, the deltoid and the supra-spinati and infra-spinati escaping in this case also. The small muscles of the hand are practically never affected. The diagnosis of these cases is founded on three things, "one is the distribution of the affected muscles, another is that there are no fibrillary contractions, and the third is the electrical reactions—that is to say, there is no reaction of degeneration. The distribution of the muscles is quite different from what you get in spinal cord lesions, or lesions of the roots of the spinal cord. With regard to the face in the Landouzy and Déjerine type the orbicularis orbis and orbicularis palpebrarum are affected, but the eye muscles are not affected—that is to say, the recti and obliqui—and the tongue is not affected. In lesions of the spinal cord and of the medulla the mouth muscles and tongue are both affected, and also the eye muscles along with the eyelids." The prognosis of these cases is much better than it is in spinal cord lesions, with the exception of pseudo-hypertrophic paralysis in which the prognosis is undoubtedly bad. With regard to pathology, the change is in the muscles and not in the nervous system at all. There is overgrowth of fat and fibrous tissue, and granular degeneration of the muscle. Whether there is apparent hypertrophy or atrophy depends on the amount of accumulation of fat which takes place. In regard to prognosis it is much more important to consider the age at which the disease comes on than the particular form it takes. When it comes on in the adult the prognosis is good, but if in early life it is much worse.

[1] Practitioner.

